# Cognitive Performance during a 24-Hour Cold Exposure Survival Simulation

**DOI:** 10.1155/2016/8130731

**Published:** 2016-07-11

**Authors:** Michael J. Taber, Geoffrey L. Hartley, Gregory W. McGarr, Dessi Zaharieva, Fabien A. Basset, Zach Hynes, Francois Haman, Bernard M. Pinet, Michel B. DuCharme, Stephen S. Cheung

**Affiliations:** ^1^Environmental Ergonomics Laboratory, Department of Kinesiology, Brock University, 500 Glenridge Avenue, St. Catharines, ON, Canada L2S 3A1; ^2^Falck Safety Services Canada, 20 Orion Court, Dartmouth, NS, Canada B2Y 4W6; ^3^Department of Human Kinetics and Recreation, Memorial University, St. John's, NL, Canada A1C 5S7; ^4^Marine and Arctic Survival Scientific and Engineering Research Team (MASSERT), Canada; ^5^School of Human Kinetics, University of Ottawa, Ottawa, ON, Canada K1N 6N5; ^6^Nutrition and Metabolism Research Unit, Montfort Hospital Research Institute, Ottawa, ON, Canada; ^7^Faculty of Health Sciences, University of Ottawa, Ottawa, ON, Canada K1N 6N5; ^8^Defence R&D Canada, Quebec City, QC, Canada G3J 1X5

## Abstract

Survivor of a ship ground in polar regions may have to wait more than five days before being rescued. Therefore, the purpose of this study was to explore cognitive performance during prolonged cold exposure. Core temperature (*T*_c_) and cognitive test battery (CTB) performance data were collected from eight participants during 24 hours of cold exposure (7.5°C ambient air temperature). Participants (recruited from those who have regular occupational exposure to cold) were instructed that they could freely engage in minimal exercise that was perceived to maintaining a tolerable level of thermal comfort. Despite the active engagement, test conditions were sufficient to significantly decrease *T*_c_ after exposure and to eliminate the typical 0.5–1.0°C circadian rise and drop in core temperature throughout a 24 h cycle. Results showed minimal changes in CTB performance regardless of exposure time. Based on the results, it is recommended that survivors who are waiting for rescue should be encouraged to engage in mild physical activity, which could have the benefit of maintaining metabolic heat production, improve motivation, and act as a distractor from cold discomfort. This recommendation should be taken into consideration during future research and when considering guidelines for mandatory survival equipment regarding cognitive performance.

## 1. Introduction

Extreme tourism (i.e., Alaskan and Antarctic cruises) is becoming more popular as larger sections of polar ice cap melt. As a result of expanding marine traffic in polar waters, there is an increase in the possibility of ships grounding on areas of previously inaccessible shoreline. Evidence of this possibility can be seen in the number of incidences reported by the National Transportation Safety Board (NTSB), the Transportation Safety Board of Canada (TSB), and various news agencies.  Table 1 shows that since the Majestic Explorer rammed into a rocky shoal on the Alaskan shoreline in 1982, leaving one passenger dead and several others injured, there have been at least 20 such events in which passengers may be faced with the possibility of evacuation into harsh environmental conditions.

It has been reported that a mass rescue operation in support of an evacuation of a cruise vessel in Arctic waters would require approximately five days to complete [1]. Although international regulations mandate the amount of food, water, and equipment International Maritime Organization (IMO) (2002): guidelines for ships operating in Arctic ice-covered waters (I:∖CIRC∖MSC∖1056-MEPC-Circ399), these supplies are only required to last for three days. Previous research, conducted over significantly shorter periods, has shown that cognitive performance is impaired by environmental thermal stress [2]. At present, it is not known whether the cognitive abilities required to perform vital survival tasks will be diminished during long-term cold exposure, thereby reducing the possibility of rescue and ultimately survival from a polar abandonment from ship or air. More specifically, it is not known whether there are measures that can be employed by the survivors to mitigate the possible deficits in performance.

Therefore, in an effort to address some of these unknown aspects of long-term survival in cool conditions, this paper presents cognitive performance findings from a 24-hour experimental cold exposure protocol in which the participants were able to actively and voluntarily control the amount of physical activity (active engagement) required to maintain a level of performance perceived to be sufficient to complete a series of cognitive tests. Based on previous research and duration of the exposure, it was expected that the cool conditions would impair some aspects of cognition (e.g., working memory and executive functioning) while having no effect or enhancing other aspects (e.g., reaction time for simple tasks).

## 2. Methods

### 2.1. Participants

The experimental protocol and instrumentation conformed to the standards set by the Declaration of Helsinki and was approved by the Research Ethics Board of Brock University (REB #09-230). Participants were medically cleared (cardiac stress test) by a primary care physician for any cardiovascular or neuromuscular symptoms and provided written informed consent prior to taking part in the study. An inclusion criterion was regular occupational or recreational experiences with cold exposure, and participants came from various professional backgrounds.

### 2.2. Experimental Design

The overriding design goal was to have participants thermally stressed to near the limits of voluntary tolerance, complete with mild hypothermia and shivering activity, for an entire 24 h without significant participant dropout (see [3], for more detail). The experimental design was developed to replicate some of the basic survival conditions that might be expected following vessel abandonment in the Arctic. For example, there was limited access to food, water, mental stimulation, and opportunities to sleep. To simulate the environmental conditions and emergency supplies that may be available in a lifeboat or life raft, no blankets or pillows were provided during the single continuous session of 24 hours of cold exposure. Participants were free to stand or sit in the chamber (described below) when they were not taking part in specific testing procedures. Prior to beginning and also following the 24-hour cold exposure, participants performed 60 min of moderate treadmill walking at 50% of their maximal aerobic capacity to simulate the level of physical exertion and activity that might be found during evacuation and rescue.

To test cognitive performance, a battery of tests that explored working and long-term memory, vigilance, absentmindedness, general mental capabilities, executive functioning, information processing ability, and spatial ability in visual working memory was presented to participants at six different time points throughout the experimental protocol (baseline, 6, 12, 18, and 24 hours of exposure, and postexposure). Upon arrival on the testing day, participants completed the cognitive test battery (CTB) as a baseline measure. After exiting the environmental chamber, participants completed a final CTB following the postexposure treadmill exercise bout. The CTB was designed to explore both simple and complex tasks in an effort to identify where, if any, deficits might occur. Thermal comfort and sensation [4] were recorded at each CTB time point.

### 2.3. Establishment of Ambient Air Temperature Protocol

Given the limited research associated with long-term exposure, ambient air temperature used for the experimental protocol was based on pilot testing. Two participants completed different components of the experimental protocol for a planned 6 hours, with one additional participant completing a full 24 hours of testing. Based on the level of experiences described by the pilot participants and previous short-term cold exposure studies [5], an ambient air temperature of 5°C was used for the pilot studies. Body core temperature data and ratings of thermal comfort were then used to identify the likelihood that participants would be able to endure/tolerate the entire 24-hour exposure. Based on the results of the pilot testing and comments from the two participants, the experimental test protocol ambient temperature was set at 7.5°C (see [3], for more detail).

### 2.4. Cognitive Testing

To reduce the likelihood of a learning effect influencing the results collected during the actual administration of the CTB, a familiarization session was conducted several days prior to the cold exposure. This familiarization session gave participants the opportunity to practice sections of the CTB and ask questions about the test procedures.

As no previous research exploring cognitive performance during prolonged cold exposure exists, specific cognitive tests were selected to explore the influence of exposure on complex information processing requirements that might be used during a survival situation. To establish a baseline measure of cognitive self-evaluation and general fluid intelligence (Gf), participants completed a cognitive failure questionnaire (CFQ) [6] and Raven's Advanced Progressive Matrices (APM) [7] ~2 hours before entering the environmental chamber. These two tests were not completed after the exposure, as it is believed that a learning effect may take place during initial administration [8]. For example, Bridger et al. [9] reported a test-retest reliability of 0.71 for CFQs completed at 12 and 36 months after the original administration of the test. Given the relatively short time period (~24 hours) between the two test periods, it was expected that the participants would remember how they had responded on the initial (pretest) CFQ and Raven's APM.

**Figure 1:**
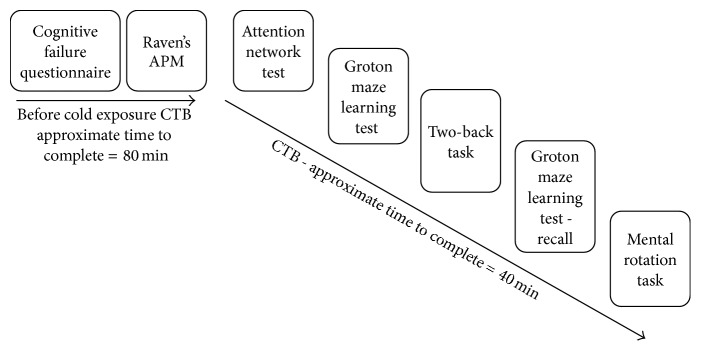
Full CTB administration protocol.

To establish a measure of progressive changes throughout the cold exposure, participants were scheduled to complete the attention network test (ANT) [10], Groton maze learning test (GMLT) [11], two-back tests (TBT), and mental rotation tasks (MRT) at predetermined intervals (6 h, 12 h, 18 h, and 24 h) beginning 60 min after initial cold exposure (Figure 1). These measures were also compared against pre/postexposure scores. The GMLT and TBT are part of the psychometric measures available from CogState [12] and have been reported to correlate well (*r*'s = 0.49–0.83) with similar psychological measures as well as show minimal practice effects [13].

This combination of tests in this integrated CTB has not been used before for cold exposure research; however, all of the measures have been examined for reliability and validity in previous cognitive performance studies [11, 14, 15]. The following description of each test outlines the areas of cognitive function believed to be important to decision making in a survival situation.

*Cognitive Failure Questionnaire (CFQ)*. The CFQ is a 25-item questionnaire that represents self-reported cognitive performance. The CFQ is measured as a total score ranging from 0 to 100 and is related to four factors of absentmindedness (memory, distractibility, blunders, and names). In addition, the CFQ items explore aspects such as spatial orientation failures, memory lapses, and motor functioning. Items are scored on a 5-point Likert scale where 0 equals “never” and 5 equals “very often.” With high CFQ scores (scored above ~45), it would be expected that participants may have considerable difficulties completing tasks that require vigilance (e.g., ANT, Groton maze, and two-back tasks) in a prolonged cold exposure environment. The CFQ has been reported to have Cronbach's alpha value of 0.91 and a test-retest reliability of 0.82 over a 2-month interval [11]. Depending on the sample, an average CFQ score may be between 19 and 45 [16].

*Raven's Advanced Progressive Matrices (APM)*. The APM is a nonverbal assessment tool designed to measure general mental capabilities pertaining to observational skills, decision-making, problem identification, perception, and sense making [15]. A total of 36 design puzzles with one piece missing are arranged in an ascending order of difficulty and raw APM scores have been used as an indicator of fluid intelligence [17, 18]. Individuals are asked to select one of four possible choices that follow puzzle design pattern rules. For the purposes of obtaining a baseline measure of cognitive ability, the APM was completed without a time limit [18].

*Attention Network Test (ANT)*. The ANT is a psychometric tool used to test the efficiency of three distinct components of the human attention network (i.e., alerting, orienting, and executive control). The test is a combination of a flanker task with arrows and a reaction time task (for a full description of the test administration methods see [10, 19]). The ANT was selected for use during the cold exposure testing as it has been shown that executive functioning is impaired when core temperatures are reduced [20]. Reliability tests for consecutive ANT performance have shown that a learning effect exists for the executive functioning, as individuals progressively get better at ignoring incongruent signals [14]. Therefore, it would be expected that if the cold exposure has no effect on cognitive functioning, reaction times should improve each time the test is administered. As the ANT was found to be too onerous during preliminary pilot testing, it was only administered during the pre-, 6-hour, 18-hour, and posttesting sessions. Ishigami et al. [21] suggest that “overall RT is itself correlated with age (*r* = 0.38), the net- work [*sic*] scores (*r* = 0.17 and 0.33 for the orienting and executive scores, resp.; ns for the alerting scores), and also with the process scores (ranging from *r* = −0.17 to −0.52; ns for processes of Divided attention and Verbal monitor- ing [*sic*])” (p. 825).

*Groton Maze Learning Test (GMLT)*. The GMLT is a computer-based neuropsychological measure (Cogstate, New Haven, CT) of working memory functioning (measured by the maze efficiency index) and information processing ability or executive functioning (measured by the number of errors) [22]. The test consists of a 10 × 10 grid of square tiles (covering a hidden pathway – 28 moves including 11 turns) presented to individuals on a touch screen computer surface. When presented to a participant, the GMLT is randomly selected from a test bank of 20 different versions of the maze, with each one equivalent in difficulty. Completing one test does not prepare the individual for subsequent GMLT, therefore, avoiding a learning effect between tests [23]. For each CTB session, the GMLT is presented six times (five initial repeated trials and one delayed recall requiring approximately 10 to 15 min to complete, which is used at the end of each of the CTB sessions to test working memory). Trials were timed (ms) and began automatically when the first move is made on the learning trial.

*Two-Back Task (TBT)*. Similar to the GMLT, the two-back task (TBT) is a computer-based measure of visual working memory and attention (CogState, New Haven, CT). The TBT presents a playing card, shown face up, in the middle of a screen. Individuals are asked to decide (select “yes” or “no”) whether a presented card is identical to one shown two cards before. An interstimulus interval of 2 seconds is used between the presentations of 35 cards. CR selected either the “d” (no) or “k” (yes) button on a standard QWERTY computer. Errors on the TBT have been reported to have a significant correlation with errors on the GMLT.

*Mental Rotation Task (MRT)*. The MRT consisted of a computer-based (available at http://bjornson.inhb.de/?p=55) test of spatial ability in which an individual is given a number of visual choices that represent a rotated version of a master image. The master image consisted of small squares arranged into a pattern presented in an upright position. Each possible choice contains the same number of small squares; however, they are arranged in slightly different patterns (with the exception of one correct choice). Difficulty in selecting the correct match to the master image is created by rotating each of the choices a specific number of degrees to the left or right (e.g., 37° or −120°). The MRT was selected to explore the capability to maintain an understanding of spatial orientation of objects, which was believed to be important during the final stages of search and rescue operations (e.g., direction of aircraft in relationship to signaling devices).

### 2.5. Data Analysis

All data were examined with IBM SPSS (version 20) software. Prior to performing the statistical analysis for hypothesis testing, the data were plotted to check for errors and outliers. Additional checks were performed to test for the assumptions of normality (Shapiro-Wilkes test) and homogeneity of variance (Levene's test). Data collected from each of the CTB were compared for each of the dependent variables in a within and between subject design. Specifically, responses for each test were compared across the different time points for each participant to identify possible changes. These responses were also combined for all participants to identify general trends in the data based on the amount of exposure time. Repeated measures analysis of variance (ANOVA) was used to explore changes in CTB score for each test. Post hoc analyses were carried out where appropriate and alpha levels were adjusted according to Bonferroni corrections.

## 3. Results

### 3.1. Initial Cognitive Assessment (CFQ and Raven's APM)

The results from the CFQ and Raven APM were used as measures of standardized cognitive processing. Due to known learning effects, these tests were only performed at baseline and were not repeated within this study. The mean CFQ score was 38.36 (SD = 8.75) (within the normal range for North American population) and all recorded CFQ scores fell within the 95% confidence interval [24]. The untimed administration of the Raven APM scores ranged from 17 to 35 with a mean of 22.9 (SD = 6.2). The scores were found to be normally distributed and based on standardized norms for a North American population [18]; the scores range from the 39th to >99th percentile.

### 3.2. Responses to Exposure

Given the intense nature of the applied research setting, participant sample size varied throughout the test protocol based on voluntary dropout. Tables 2 and 3 provide participant demographic information as well as an overview of previous occupational or recreational exposure to cold environments. Although the exclusion criteria were designed to limit the dropout rate, these fluctuations in sample size reflect situation in which it might be expected that within a given population forced to evacuate a commercial airliner or cruise ship some individuals may not survive until rescue arrives.

Given that the participants were recruited for their past experience, Table 3 details the relevant cold exposure information. From the table it can be seen that on average the participants have more than 18 years of cold exposure experience in temperatures ranging from −15°C to −56°C.

*Subject Rating of Difficulty*. On a subjective rating of difficulty where zero represented not difficult at all and 10 represented extremely difficult/need to withdraw from the study, participants rated the experience as an 8. Seven of the eight (88%) individuals indicated that there was at least one point throughout the trial that they believed they would have to voluntarily withdraw from the testing. Two of the participants voluntarily removed themselves from the experimental protocol—one at 6.5 h and the other at 13 h. CTB results include the scores of these two participants for the period in which they remained in the trial (i.e., during the baseline and first 12 hours). Another participant performed the entire 24 hours but required the use of the thermal blanket from 16 h onwards. Overall, this suggests that the conditions were sufficiently taxing physically and mentally even for this motivated and self-selected participant pool. Seven of eight participants reported minimal sleep (~1.5 h) over the 24 hours. None of the participants found the bedspace to be a safe haven or comfortable, and most declined to use the bedspace or prematurely removed themselves from it over the course of cold exposure.

*Core Temperature (T*_c_). No participants were removed from the experiment due to core temperature reaching 35.0°C, though two participants used the thermal blanket at various points throughout the testing. For most participants, core temperature generally decreased ~0.6°C (SD = 0.3) within the first 12 h of exposure and then stabilized at that approximate level for the remainder of the 24-hour exposure. Fluctuations within a range of 0.5°C occurred during this latter “stable” period, but overall the participants were able to sufficiently thermoregulate through shivering and some active engagement of mild exercise. Overall, this drop in core temperature was found to be significant (*F*_(4,32)_ = 6.99, *p* < 0.001), with a ~0.4°C (SD = 0.4) drop in core temperature between the baseline (pretest) and all other time points (6, 12, 18, and 24 hours of exposure). A similar examination of both thermal comfort and sensation did not reveal any significant differences across the 24 hours of exposure. Importantly, the thermal exposure also eliminated the typical 0.5–1.0°C circadian rise and drop in core temperature throughout a 24-hour cycle [25], such that the true level of hypothermic strain exceeded the ~0.4°C absolute *T*_c_ decrease for much of the exposure.

*ANT Results*. The individual mean scores of the three networks: alerting, orientation, and conflict, were 32.9 ms (SD = 18.3), 53.0 ms (SD = 23.9), and 138.1 ms (SD = 31.4), respectively. The mean total reaction time for correct trials was 652.5 ms (SD = 64.9). The test was administered at four different time point: before cold exposure; 6 hours; 18 hours, and after cold exposure. A one-way ANOVA indicated that there were no significant differences between scores based on administration time for alerting, orientation, or conflict. No significant differences in total mean reaction time were found for any of the test blocks.

**Figure 2:**
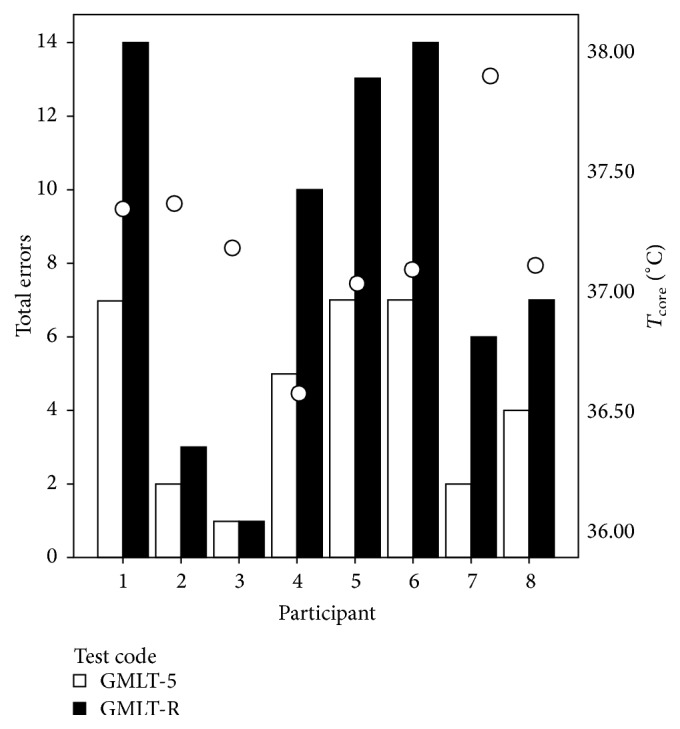
Total GMLT error based on GMLT-5 and GMLT-Recall during the postexposure session. The white circles represent the mean *T*_c_ of participants at the time of completing the recall GMLT.

*GMLT Results*. As part of the computerized CogState portion of the CTB, the GMLT was completed a total of six times during the course of this study (before, 6 h, 12 h, 18 h, 24 h, and after). Given the test protocol, individual GMLT were presented to the participant seven times (initial test sequence, five consecutive presentations, and one recall presentation after completing the TBT). After the initial presentation, the final maze in the initial block (Test Code GMLT-5) error rate was compared with the recall maze (Test Code GMLT-R) using a Wilcoxon Signed Rank Test. No significant changes in performance were noted across the trials. However, when comparing the same two presentations of the Groton maze for the postexposure session, the results indicate that there was a significant difference between the number of errors committed (*Z* = −2.37, *p* = 0.018).  Figure 2 shows that almost all of the participants committed more errors while completing the recall GMLT during the postexposure CTB session. No other significant findings were found.

*TBT Results*. The two-back task was administered during the same time points as the GMLT. A repeated measures ANOVA was conducted to explore the TBT speed, variability, accuracy, and number of errors. No significant findings were found regardless of trial administration time.

*MRT Results*. The mental rotation task (MRT) measured the accuracy, speed, errors, and time for the 10 images within each test block.  Table 4 displays the mean reaction time (RT) and accuracy for each of the test blocks.

**Figure 3:**
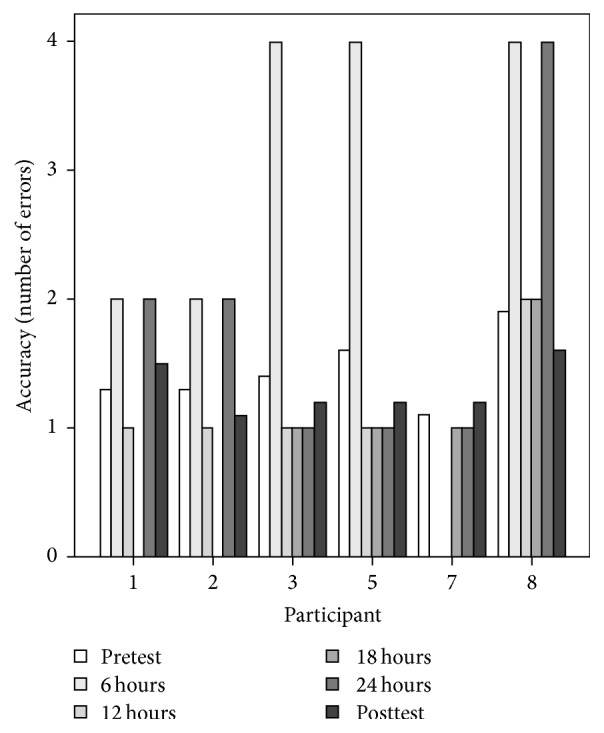
MRT errors committed by each participant across all trials.

 A Friedman Test revealed that there was a significant reduction in MRT accuracy (*n* = 6, *p* = 0.04). Post hoc analyses indicated that the significant difference occurred between first test after beginning the exposure (6 h) and after 18 h of exposure (Figure 3). Posttest results indicate that when completing the computer-based MRT, participants did not require significantly more time or commit more errors when compared to the pretest MRT. Results further indicated that pretest and posttest computer-based MRT correlate well with one another (*r*(6) = 0.738, *p* = 0.037). No other significant findings were found for speed or accuracy regardless of the time at which the test was completed.

### 3.3. Combined Cognitive Effects

This section of the results addresses some of the combined effects of the experimental conditions on cognitive performance. Correlation analyses reveal that there were several significant relationships that existed within the CTB results; however, no effect of time was found for performance.  Table 5 displays the correlation table for all of the tests as they relate to one another.

## 4. Discussion

This study aimed to simulate a prolonged survival scenario that might occur following a ship or plane incident in a cold environment. The primary goal was to elicit a sustained moderate thermal stress throughout 24 h, including a decrease in core temperature and elevation in metabolism through shivering. Additionally, we simulated many of the attendant situational factors, including isolation, boredom, food quality and availability, and sleep restriction. Rather than the restricted movement or voluntary physical activity in traditional hypothermia research, we permitted self-engaged physical activity within the confines of the testing chamber to replicate what might occur in a survival scenario. Overall, despite these challenging experimental conditions (confirmed by the participant difficulty ratings), cognitive performance (measured by the CTB) did not significantly alter throughout the course of the prolonged 24 h of cold exposure compared to baseline values taken before cold exposure. Together, this suggests that cognitive performance may be maintainable through sustained cold exposure, assuming that severe hypothermia can be avoided.

Despite previous cold exposure research showing a decrement in simple and choice serial reaction times, memory, sustained vigilance, and target tracking [26–28], others have shown that little or no changes in cognitive performance will occur over prolonged exposure if individuals are given the opportunity to self-regulate the amount of protective clothing worn or where exercise is used during the exposure sessions. For example, Slaven and Windle [29] showed that there were no significant decreases in cognitive performance in serial RT, Sternberg one-letter or seven-letter recall accuracy or speed. These findings were based on four days of consecutive testing in which the ambient air temperature was 15°C, 5.8°C, 4.4°C, and 4.4°C (resp.) and participants were able to select between three options of thermal protection. Similarly, Banderet et al. [30] found that over a five-day cold exposure session (ambient air temperatures ranged from −4°C at night to −25°C during the day) which included physical activity, significant differences in cognitive performance were only found for individuals who were hypohydrated at or below 2.5% of total body weight. In addition to the Slaven and Windle [29] and Banderet et al. [30] studies within an applied setting, Baddeley et al. [31] suggest that the lack of changes in cognitive performance found during exposure to 4.4°C water for approximately 60 minutes was due to highly motivated divers. Exposure times for all of these studies were considerably less than those carried out in this examination of cognitive performance.

Flouris et al. [28] show deterioration of vigilance and reaction time within the first 45 minutes of exposure to −20°C ambient air temperature, while a meta-analysis of cold exposure studies revealed that cognitive performance is decreased by an average of 14% in temperature at 10°C or less [32]. Meta-analyses [32, 33] have identified thermally induced reductions in cognitive performance that are most often observed when tasks are highly complex, require sustained vigilance, and place a considerable load on working memory. However, within these studies, the decrements to cognitive performance have been limited to specific domains such as working memory and vigilance [22], while effects have also been shown in long-term memory recall [26].

The disparity in research findings on the effects of cold-induced changes in cognitive performance has previously been explained by suggesting that the environmental stimuli (hot or cold ambient temperatures) act as a distractor [27, 34, 35] or as form of arousal [36, 37]. Based on the results from each of the cognitive tests, it appears that cognition was not significantly affected when examining overall changes during the long-term exposure. Given the paucity of thermoregulatory research assessing cognitive performance in long-term (more than 6 hours) cold exposure, it is interesting to note that Pilcher et al. [32] and Pietrzak et al. [22] reported that high intensity/short-term exposure had a greater negative influence on performance than less intense/long-term exposure during testing. These findings have been supported by research suggesting that cognitive performance above ambient temperatures of approximately 11°C will have minimal or no changes, whereas ambient conditions below this temperature result in deleterious effects [33]. Finally, Færevik et al. [38] reported that minimal physical activity can be used to minimize a reduction in core temperature over long-term cold exposure (24 hours). Together, this suggests that there exists a zone of optimal cognitive performance from maintaining thermoneutrality, similar to that suggested by Hanin [36] and the extended-U hypotheses proposed by Hancock and Warm [37]. Both models suggest that there is a specific zone in which individuals will perform at maximal levels; however, performance becomes degraded if individuals are expected to perform tasks outside this optimal zone. Thermal stressors that do not increase the level of arousal beyond the optimal zone should, therefore, not be expected to adversely affect the performance of skills that are well rehearsed or require minimal cognitive effort.

One explanation for the limited changes to cognitive performance in this study is believed to be the amount of mild exercise performed by the participants and it was noted that individuals generally stood throughout the entire experimental protocol, indicating that it was too cold to sit for any length of time. It was observed throughout the cold exposure trials that participants would engage in mild physical activity such as hopping in one spot, swinging arms around their torso in a hugging motion, or vigorously rubbing their limbs after every test session that required them to sit for any period of time. Færevik et al. [38] showed that minimal exercise in cold conditions affects core temperature, suggesting that “5-min periods of moderate cycling leg movements every 20 min reduced shivering intensity, improved heat balance, slowed core cooling, and had a positive effect on the subjective perception of thermal comfort and reduced cold sensation” (p. 1000). Specifically, in an effort to explore the effects of exercise on body core while in water, Færevik et al. [38] reported that, in −2°C ambient air, 2°C water with 30–40 cm waves, participants rate of core cooling was significantly less when they performed moderate (sustainable) cycling for 5 minutes every 20 minutes. In fact, it was reported that not only did the moderate exercise decrease the rate of core cooling, it also significantly increased heat production to the point that a 10% gain was observed [38].

A second explanation for the results may be related to the experimental design of the project and appear to support previous research suggesting that minimal or no differences in cognitive performance should be expected when the intensity of the stressor (the cold ambient air in this case) is low [3, 22, 32, 33]. These findings also appear to support the results reported by Slaven and Windle [29] in which they describe that choice RT tests and short-term memory were unaffected after seven days in a simulated submarine in distress at 4.4°C. Similarly, Giesbrecht et al. [39] showed no significant difference in the performance of simple tasks based on cold exposure. Enander [27] noted that there were no decrements in simple RT tasks when participants were exposed to 5°C ambient air temperature over a period of 55 to 90 minutes (see also [39]). Participants in this prolonged cold exposure study were given the opportunity to engage in any activity that would help them stay warm enough to endure the full 24-hour exposure protocol. Despite the initial drop in core temperature, this active engagement potentially provides a coping mechanism during the testing and possibly could equate to a survival advantage in abandonment. There did not appear one definitive change in cognitive performance over the course of the experimental session. For example, there was no difference in attention related results (ANT and TBT), while there was a minor (nonsignificant) shift (more errors) in the working memory after 12 hours of exposure, and MRT results showed that, at 6 and 24 hours, there was a tendency for the participants to require more time to complete the questions and committed more errors (also not found to be significant).

Finally, it could be argued that another explanation for the findings is related to the level of stimulation present through the experimental session. The results might suggest that the level of arousal associated with the cold ambient air, constant shivering, cognitive testing, limited sleep, and confined conditions fell within an optimal zone of functioning for the selected group of participants [36, 37]. The only exception to this argument of the conditions falling within the optimal zone was found for the MRT errors. It is possible that the significantly higher number of errors in the MRT (when compared to the performance at 18 hours of exposure) could suggest that the level of arousal in the initial part of the testing was sufficient to influence the performance. As previously mentioned, the group of participants was specifically selected for this study to ensure a high success rate of completion. It may be that the intensity of the experimental protocol was ideally suited to provide a tolerable level in which performance was not affected [40]. For example, the ANT results were consistent with values reported by Weaver et al. [41] who found an overall mean reaction time (RT) of 646.5 (SD = 128.4), an alerting mean score of 33.0 (SD = 46.4), orienting mean score of 42.4 (SD = 37.4), and conflict mean score of 163.5 (SD = 90.0).

The results indicated that there were significant correlations between a number of the CTB measures. Given the specific cognitive tests used in this study and the reported validity, it would be expected that there would be strong correlations between and within particular components of the CTB. It was, however, somewhat unexpected that no changes occurred in the latter portion of the prolonged exposure. Given the fact that no time related correlations (e.g., negative relationship) were found, it can be assumed that the participants were sufficiently stimulated to overcome the expected influence on fatigue.

### 4.1. Main Contributions

As the majority of previous cold exposure research is conducted over considerably shorter periods of time (e.g., <6 hours), the primary contributions from this study are related to the extension of cognitive performance data over a much longer time frame. Additionally, the novel experimental protocol, which allowed individuals to actively engage in mild exercise, situates the data in more realistic conditions. For example, unless injuries preclude movement and assuming normothermic core temperatures as seen in this study, it is unlikely that survivors of a vessel abandonment will passively sit in one position while they continue to cool to the point that they no longer have the capability to help themselves.

### 4.2. Limitations

With no other changes despite a slight decrease in core temperature, constant shivering throughout the exposure period, lack of sleep, and minimal food, it could be argued that it would be difficult to explain which factor(s) allowed participants to remain at a nearly constant level of cognitive performance. The limited number of participants tested in this study and the changes, both positive and negative depending on the type of measure, may have been due to fatigue or exposure or a combination of several other factors. Additionally, the individual differences in the responses to the GMLT may have obscured the effects of the cold response. Significant changes may also have been mediated by increased arousal levels associated with the distractive nature of the cold exposure [26].

### 4.3. Conclusions

In summary, despite a realistic survival simulation involving 24 hours of prolonged cold exposure, moderate decreases in core temperature, and sustained shivering, cognitive performance was largely maintained. This suggests that, as long as significant hypothermia is prevented, survivors may be capable of maintaining a range of simple through complex cognitive tasks for at least the first 24 hours of abandonment. One potential contributor to this performance maintenance may be the allowance of mild, self-engaged physical activity, which could have the dual benefit of maintaining core temperature and also improving motivation and acting as a distractor from the cold discomfort.

## Figures and Tables

**Table 1:** Cruise ship emergency events by region.

Name of vessel	Year	Region	Evacuation method
Majestic explorer	1982	Arctic	Inflatable liferafts
Nieuw Amsterdam	1994	Arctic	Refloated
Star princess	1995	Arctic	Evacuated to another ship
Spirit of 98	1999	Arctic	Inflatable liferafts
Wilderness explorer	1999	Arctic	Refloated
Clipper adventure	2002	Antarctica	Freed by Chilean icebreaker
Mona lisa	2003	Arctic	Evacuated to another ship
Le conte	2004	Arctic	Evacuated to another ship
Wilderness adventurer	2004	Arctic	Evacuated to another ship
Clipper odyssey	2004	Arctic	Coast Guard assistance
Lyubov orlova	2006	Antarctica	Transferred to another ship
Nordkapp	2007	Antarctica	Transferred to another ship
MV explorer	2007	Antarctica	Lifeboats
Empress of the north	2007	Arctic	Coast Guard assistance
Spirit of Alaska	2008	Arctic	Coast Guard assist/transferred to another ship
Spirit of glacier bay	2008	Arctic	Evacuated to Coast Guard vessel
Ushuaia	2008	Antarctica	Evacuated to Chilean navy vessel
Antarctic dream	2008	Antarctica	Free by a research vessel
Ocean nova	2009	Antarctica	Evacuated to Argentine Navy vessel
Clipper adventurer	2010	Arctic	Coast Guard assistance
Clelia II	2010	Antarctica	Assisted by NG Explorer
Polar star	2011	Antarctica	Evacuated to Argentine Navy vessel
Sea spirit	2013	Arctic	Zodiac capsizes during shore excursion
Silver explorer	2013	Antarctica	Damaged by 18' large wave

**Table 2:** Participant demographic and physiological information.

Participant	Measure							
	Age	Height (cm)	Weight before (kg)	Weight after (kg)	% body fat	USG before	USG after	$\dot{V}O_{2max}$ (mL/min/kg)
1	47	178.5	103.6	103.42	23.57	1.014	1.019	30.6
2	22	180	89.96	88.9	12.6	1.003	1.018	57
3	44	175.5	92.48	90.82	13.03	1.006		53
4	25	186.5	114.7	113.5	17.5	1.003		42.2
5	35	181.8	132.78	130.5	20.90	1.013	1.015	40.8
6	30	179.5	87.02	86.18	12.54	1.005		55.5
7	22	178	88.3	86.62	9.92	1.005	1.002	55.8
8	35	175.5	81.84	80.02	13.37	1.005	1.019	60
Mean	32.5	179.41	98.83	97.50	15.43	1.006		49.36
(SD)	(9.55)	(3.58)	(17.26)	(17.12)	(4.74)	(0.004)		(10.29)

**Table 3:** Participant cold exposure experience.

Participant	Cold exposure experience			
	Years of experience	Coldest temperature (°C)	Duration of exposure (hours)	Last exposure
1	25	−56	11	Within last 6 months
2	5	−20	6	Within last 6 months
3	20	−15	8	Within last 6 months
4	24	−45	6	Within last 6 months
5	27	−30	5	Within last 12 months
6	15	−25	1	Within last 6 months
7	15	−30	12	Within last 6 months
8	Did not respond to questionnaire			
Mean	18.71	−31.57	7	
(SD)	7.67	14.34	3.74	

**Table 4:** Mean (SD) reaction times and accuracy for the MRT based on test blocks.

Test score	Test block (hour)					
	Before	6	12	18	24	After
Reaction time (ms) (SD)	8.6 (2.5)	9.1 (3.6)	8.4 (2.1)	7.5 (1.0)	9.7 (2.8)	7.4 (1.8)
Accuracy (number of errors)	1.4 (0.3)	2.7 (1.6)	1.0 (0.6)	0.8 (0.8)	1.8 (1.2)	1.3 (0.2)

**Table 5:** Correlation table for CTB administration.

Correlations									
	CFQ	APM	Alerting	Orientation	Conflict	ANT RT	GMLT errors	MRT speed	MRT accuracy
CFQ score									
Pearson correlation	1								
Sig. (2-tailed)									
*N*	8								
APM score									
Pearson correlation	0.132	1							
Sig. (2-tailed)	0.755								
*N*	8	8							
ANT alerting									
Pearson correlation	−0.060	0.766^*∗*^	1						
Sig. (2-tailed)	0.888	0.027							
*N*	8	8	29						
ANT orientation									
Pearson correlation	0.233	−0.195	0.249	1					
Sig. (2-tailed)	0.579	0.643	0.193						
*N*	8	8	29	29					
ANT conflict									
Pearson correlation	0.397	0.382	0.081	0.356	1				
Sig. (2-tailed)	0.330	0.350	0.674	0.058					
*N*	8	8	29	29	29				
ANT reaction time									
Pearson correlation	0.131	0.072	−0.036	0.587^*∗∗*^	0.750^*∗∗*^	1			
Sig. (2-tailed)	0.757	0.865	0.853	0.001	0.000				
*N*	8	8	29	29	29	29			
GMLT total errors									
Pearson correlation	0.477	−0.180	0.434^*∗*^	0.298	0.089	0.194	1		
Sig. (2-tailed)	0.279	0.700	0.021	0.124	0.653	0.323			
*N*	7	7	28	28	28	28	319		
MRT speed									
Pearson correlation	0.447	0.062	0.031	0.332	0.150	0.218	0.295	1	
Sig. (2-tailed)	0.267	0.884	0.877	0.091	0.454	0.274	0.064		
*N*	8	8	27	27	27	27	40	43	
MRT accuracy									
Pearson correlation	0.775^*∗*^	−0.244	−0.126	0.160	−0.076	−0.132	0.138	0.451^*∗∗*^	1
Sig. (2-tailed)	0.024	0.560	0.531	0.425	0.705	0.511	0.396	0.002	
*N*	8	8	27	27	27	27	40	43	43

^*∗*^Correlation is significant at the 0.05 level (2-tailed).

^*∗∗*^Correlation is significant at the 0.01 level (2-tailed).
